# Whole genome sequencing characteristics of *Chlamydia psittaci* caprine AMK-16 strain, a promising killed whole cell veterinary vaccine candidate against chlamydia infection

**DOI:** 10.1371/journal.pone.0293612

**Published:** 2023-10-30

**Authors:** Valentina A. Feodorova, Sergey S. Zaitsev, Anna M. Lyapina, Natalya V. Kichemazova, Yury V. Saltykov, Mariya A. Khizhnyakova, Vitaliy V. Evstifeev, Olga S. Larionova

**Affiliations:** 1 Laboratory for Fundamental and Applied Research, Department for Microbiology and Biotechnology, Saratov State University of Genetics, Biotechnology and Engineering Named After N.I. Vavilov, Saratov, Russia; 2 Laboratory for Fundamental and Applied Research, Saratov State University of Genetics, Biotechnology and Engineering Named After N.I. Vavilov, Saratov, Russia; 3 Laboratory of Viral and Chlamydial Infections, Federal Center for Toxicological, Radiation and Biological Safety, Kazan, Russia; 4 Department of Microbiology, Virology and Immunology, Kazan State Academy of Veterinary Medicine by N.E. Bauman, Kazan City, Russia; Kafrelsheikh University Faculty of Veterinary Medicine, EGYPT

## Abstract

*Chlamydia psittaci* is a primary zoonotic pathogen with a broad host range causing severe respiratory and reproductive system infection in animals and humans. To reduce the global burden of *C*. *psittaci*-associated diseases on animal welfare and health and to control the pathogen spread in husbandry, effective vaccines based on promising vaccine candidate(s) are required. Recently, the caprine *C*. *psittaci* AMK-16 strain (AMK-16) demonstrated a high level of protection (up to 80–100%) in outbred mice and pregnant rabbits immunized with these formaldehyde-inactivated bacteria against experimental chlamydial wild-type infection. This study investigated the molecular characteristics of AMK-16 by whole-genome sequencing followed by molecular typing, phylogenetic analysis and detection of main immunodominant protein(s) eliciting the immune response in mouse model. Similarly to other *C*. *psittaci*, AMK-16 harbored an extrachromosomal plasmid. The whole-genome phylogenetic analysis proved that AMK-16 strain belonging to ST28 clustered with only *C*. *psittaci* but not with *Chlamydia abortus* strains. However, AMK-16 possessed the insert which resulted from the recombination event as the additional single chromosome region of a 23,100 bp size with higher homology to *C*. *abortus* (98.38–99.94%) rather than to *C*. *psittaci* (92.06–92.55%). At least six of 16 CDSs were absent in AMK-16 plasticity zone and 41 CDSs in other loci compared with the reference *C*. *psittaci* 6BC strain. Two SNPs identified in the AMK-16 *ompA* sequence resulted in MOMP polymorphism followed by the formation of a novel genotype/subtype including three other *C*. *psittaci* strains else. AMK-16 MOMP provided marked specific cellular and humoral immune response in 100% of mice immunized with the inactivated AMK-16 bacteria. Both DnaK and GrpE encoded by the recombination region genes were less immunoreactive, inducing only a negligible T-cell murine immune response, while homologous antibodies could be detected in 50% and 30% of immunized mice, respectively. Thus, AMK-16 could be a promising vaccine candidate for the development of a killed whole cell vaccine against chlamydiosis in livestock.

## Introduction

*Chlamydia psittaci* is Gram-negative, an obligate intracellular pathogen, one of the 14 characterized species of the family *Chlamydiaceae* [1–3]. *C*. *psittaci* is globally recognized as the causative agent of a number of infectious diseases in animals, humans, poultry, reptiles, wild and decorative pet birds, especially parrots [1–5]. Initially, these bacteria were associated with only avian psittacosis [4–6]. However, since then it has been clearly proven that *C*. *psittaci* is also responsible for airway inflammation and reproductive disorders in cattle, small ruminants, domestic animals and humans [7–13]. *C*. *psittaci* (ovis), which is now re-classified to *Chlamydia abortus*, is responsible for abortion, stillbirth or fetal loss in ruminants worldwide. Small ruminants, sheep, and goats infected with this pathogen, may develop chlamydiosis, which is also known as ovine enzootic abortion (OEA) or enzootic abortion of ewes (EAE) [14]. OEA is a common life-threatening infection in small ruminants whose outbreaks may cause global financial losses to the world animal husbandry industry. Therefore, each case of the disease demands an obligatory notification to the World Organization for Animal Health (WOAH) (https://www.woah.org/).

Both organisms, *C*. *psittaci* and *C*. *abortus*, are pathogenic to humans. As a rule, human infection is a result of inhaling the infected material derived from some zoonotic source, namely sick or infected livestock or birds. This can lead to animal and human respiratory diseases. Chlamydia infection in pregnant women may develop severe, sometimes life-threatening, outcomes in a mother herself, as well as result in stillbirth or miscarriage [15–17].

The organisms, *C*. *psittaci* and *C*. *abortus*, may establish in sensitive hosts either chlamydia monoinfection individually associated with each of these pathogens or simultaneous mixed infection caused by both chlamydia species [11, 18–21]. Cases of chlamydia infection in animals caused by chlamydia strains associated with recombination events in chromosomes were detected more often in *C*. *psittaci* than in *C*. *abortus* and were also reported for other *Chlamydia spp*. [22–25].

Virulence-attenuated or fully virulent strains of either *C*. *psittaci* or *C*. *abortus* have been actively tested for the development of either live or killed whole-cell vaccines (LWCV or KWCV, respectively) for prophylaxis of chlamydiosis in farm animals. However, recently a dramatic number of marked postvaccinal complications known as ’abortion storm’ [26] has been registered in ewes after their immunization with the commercially available LWC vaccine based on the attenuated mutant *C*. *abortus* strain 1B. Additionally, this strain was proven to be transmitted from vaccinated to naïve unvaccinated animals, which resulted in the development of clinical features of a natural chlamydial wild-type infection [26–29]. In fact, KWCVs intended for the prophylaxis of chlamydia in farm animals have been developed and successfully commercialized [30, 31]. This type of vaccines led to a decrease in chlamydia-associated abortion cases and absolutely abolished any vaccine-related complications such as chlamydia infection of the immunized sheep, transmission and uncontrolled spread of the relevant live vaccine strain from a vaccinee to naïve farm animals. Nevertheless, the efficacy of KWCVs remains questionable [32–36]. This is a critical point in the development of effective chlamydia KWCVs for veterinary use, which highlighted the great importance of the evaluation of novel alternative vaccine candidate(s) based on wild virulent chlamydia strains. This could implicate the need to investigate the main genome characteristics of chlamydia strains recently derived from ruminants with chlamydiosis if those inactivated virulent isolates demonstrate a marked protective potency against experimental chlamydial infection.

Recently, it has been shown that immunization of small animal models (outbred mice and pregnant rabbits) with the formaldehyde-inactivated *C*. *psittaci* AMK-16 strain could induce a protection against model chlamydia wild-type infection. The immunized animals developed a detectable level of humoral response registered in the complement fixation test (CFT) with the use of the whole bacterial suspension as the antigen [37]. However, neither the role of individual proteins in the elicited antibody response nor the cellular immune reactions were investigated in a well-characterized murine model widely used for immunogenicity research [30, 31, 36–47].

Therefore, the main aim of this study was to sequence and investigate the main molecular characteristics of the *C*. *psittaci* AMK-16 strain genome in order to better understand: (i) the bacterial diversity of *C*. *psittaci* strains which currently cause chlamydia infection in small ruminants; and (ii) T- and B-cell immune response to the AMK-16 immunodominant proteins elicited by the immunization with the AMK-16 inactivated bacteria in a mouse model.

## Materials and methods

### Bacterial strains

The Chlamydia strains used in this study are listed in S1 Table in S1 File. The *C*. *psittaci* AMK-16 was initially isolated in 2016 from the fetus of the aborted dairy goat under a local chlamydia outbreak in the private household in the Middle Volga Region of Russia. For whole-genome sequencing and immunological studies, the strain was routinely propagated in the yolk sac of chicken embryos following purification through Renografin density gradient centrifugation as previously described [6, 22]. The strain was kept either frozen at -70° C or through lyophilization as reported [48, 49].

### DNA extraction and whole-genome sequencing

The total DNA was isolated from the AMK-16 lyophilized suspension using a commercial kit (DNA DNeasy Blood & Tissue Kit, Qiagen, Germany) according to manufacturer’s instructions. The concentration of the extracted DNA was routinely measured using a spectrophotometer (Bio-Rad, USA). The DNA was stored at −20° C until further use.

The AMK-16-derived DNA was sequenced in parallel on two sequencing platforms (Illumina and Nanopore). In the first case, the DNA was sequenced as 2×150 bp paired-end reads on the Illumina HiSeq 2500 platform (Illumina Inc., USA) at the Genoanalytica LLC (https://www.genoanalytica.ru/, Moscow, Russia). For this purpose, DNA library was performed using Nextera XT DNA Library Preparation Kit (Illumine Inc., USA). Preparation of DNA library for Nanopore MinION sequencing was performed using Kit SQK-LSK109 (Oxford Nanopore, UK). The standard protocol was applied according to the manufacturer’s recommendations (https://nanoporetech.com/). A FLO-MIN-106 R9.4 Flow cell (Oxford Nanopore Technologies, Oxford, UK) was routinely used for sequencing with the MinION and the MinKNOW software as recommended (https://nanoporetech.com/). Hybrid assembly of the AMK-16 genome by the *de novo* method was generated with Unicycler v0.4.9. (https://github.com/rrwick/Unicycler). SRA accession number was SRS16946381 on NCBI.

The annotation of this genome was performed by the NCBI Prokaryotic Genome Annotation Pipeline (PGAP) (https://www.ncbi.nlm.nih.gov/genome/annotation_prok/) [50]. The complete genome of the AMK-16 strain was deposited at the NCBI GenBank (Acc. number for the chromosome is CP047319.1, and for the relevant plasmid, pAMK complete sequence—CP047320.1, S1 Table in S1 File).

Construction of the phylogenetic tree for the *C*. *psittaci* and *C*. *abortus* strains was performed using the MEGA–7 software [51] by the maximum likelihood method. For this purpose, *C*. *psittaci* strains (n = 27) and *C*. *abortus* strains (n = 9) with the status of “complete assembly level” available in the NCBI GenBank database (https://www.ncbi.nlm.nih.gov/genbank/, accessed on 06 June 2023) were selected. Protein sequence-based genome comparison of AMK-16 and reference GR9 strains was performed using Proteome Comparison Service tool on The Bacterial and Viral Bioinformatics Resource Center (BV-BRC) platform [52].

Whole genome phylogenetic analysis was done on The Bacterial and Viral Bioinformatics Resource Center (BV-BRC) using default settings [52]. Recombination analysis and construction of phylogenetic trees were carried out using RDP4.101 software (http://web.cbio.uct.ac.za/~darren/rdp.html) [53] with Automated MaxChi method.

### Multi-locus sequence typing (MLST)

Multi-locus sequence typing (MLST) of the *C*. *psittaci* AMK-16 strain based on the concatenated fragments of seven housekeeping genes (*gatA*, *oppA*, *hflX*, *gidA*, *enoA*, *hemN* and *fumC*) was performed as previously described [22, 54, 55]. The relevant sequence type (ST) for the AMK-16 was assigned by comparison with the allelic profiles for the *Chlamydia* strains available through the MLST online database [56, 57] for *Chlamydiales* (http://pubmlst.org/chlamydiales/). The MLST-derived allele sequences of the AMK-16 were deposited in PubMLST (https://pubmlst.org/) with the access number/ID 4920. The minimum spanning tree based on the concatenated nucleotide sequences of seven house-keeping genes was constructed and visualized with the GrapeTree to reveal the phylogenetic relationships between the *C*. *psittaci* AMK-16 and other *C*. *psittaci* strains (n = 224) available in the PubMLST database (https://pubmlst.org/, accessed on 06 June 2023) (S2 Table in S1 File).

### *ompA* typing

The AMK-16 genotyping was applied to the full-length major outer membrane protein (*ompA*) gene sequencing derived from the relevant whole genome sequence available in the NCBI GenBank (Acc. number: CP047319.1, the locus tag GR632_02940). Phylogenetic analysis based on the *ompA* reference sequences derived from the ten *C*. *psittaci* classical genotypes (A—F, E/B, Mat116, M56 and WC) (S1 Table in S1 File) and the AMK-16 *ompA* sequence was performed with the use of the MEGA–7 software [51] by the maximum likelihood method.

### Animal models and immunization

#### Mice

For immunization experiments the formalin-inactivated lyophilized *C*. *psittaci* AMK-16 bacterial suspension was diluted in sterile phosphate-buffered saline (PBS), and the total protein concentration of the resulting solution was determined by the Bradford assay. Six- to eight-week-old male outbred mice (n = 6) were immunized twice intraperitoneally at a 14-day interval with this *C*. *psittaci* AMK-16 bacterial suspension. The optimal immunization dose was determined in preliminary experiments and has been established as 20 mkg of protein per mouse. The inoculum was mixed 1:1 (v/v) with complete Freund’s adjuvant (CFA) (Sigma, USA) for the first injection and incomplete Freund’s adjuvant (IFA) (Sigma, USA) for the second immunization.

The control group of mice (n = 7) was inoculated with PBS mixed with CFA or IFA according to the same schedule. On the day 7 after the second injection all mice were sacrificed by blood withdrawal via cardiac puncture under the zolazepam-tiletamine plus xylazine terminal anesthesia, and spleens and cardiac blood were obtained for lymphocyte proliferation and serological assays, respectively. All animal procedures were carried out in strict accordance with the National Regulations. The animal study protocol was approved by the Institutional Review Board of the State Educational Institution of Higher Professional Education, Saratov Medical State University (IRB#00005197) and followed the rules stated in the Declaration of Helsinki.

#### Ewes

Naïve 6-8-month-old ewes (n = 8) were immunized *i*.*m*. three times with the inactivated bacteria of the *C*. *psittaci* homologous strain as described earlier [34]. Sera were obtained from blood samples, lyophilized and stored for future investigations.

### Evaluation of immune response elicited by the *C*. *psittaci* AMK-16 strain in animal models

#### Evaluation of humoral murine and ovine immune responses in immunoblotting

Sera collected from either mice or ewes immunized with the inactivated AMK-16 bacterial suspension were analyzed by immunoblot technique as was described previously [38], with the panel of the purified either recombinant or chemically isolated proteins including: (i) the MOMP derived from the AMK-16 (MOMP AMK-16) and the MOMP of the wild type *C*. *psittaci* GR9 reference strain (MOMP- GR9); (ii) the DnaK and the GrpE of the either AMK-16 or the *C*. *psittaci* GR9 (DnaK AMK-16, DnaK GR9, GrpE AMK-16 and GrpE GR9, respectively); (iii) the MOMP-containing C-antigen isolated from embryonated chicken eggs-derived (ECEs) suspension of chlamydia by a routine technique [6, 22] after inoculation to ECEs of the *C*. *psittaci* AMK-16 (C-ag AMK-16). In addition, the whole-cell lysate of the *C*. *psittaci* AMK-16 suspension inactivated by heat exposure [6, 22, 37] was used. All recombinant antigens were ordered from AtaGenix Laboratories (China, http://en.atagenix.com).

Briefly, the antigens and bacterial lysate were resolved by electrophoresis in 12.5% SDS-PAGE [58], and the proteins were immediately transferred to a 0.22 μm pore size nitrocellulose membrane (Bio-Rad, USA). The membranes were blocked with 1% nonfat dried milk in PBS at room temperature for 1 hour with middle agitation. Then the membranes were washed twice in PBS and 0.05% Tween-20 (PBST) and incubated overnight at 4°C with either murine or ovine sera diluted in 1% nonfat dried milk/PBST at the ratio 1:100 or 1:10–1:40, respectively. After four washings with PBST, the membranes were probed with either HRP-conjugated goat anti-mouse or mouse anti-goat/sheep IgG (both Sigma, USA) at the dilution 1:25000 or 1:14000, respectively. Then, the washing step was repeated five times, and the reaction was developed with chromogenic substrate solution (3,3,5,5′-Tetramethylbenzidine (TMB) liquid substrate system for membranes, Sigma, USA).

#### Evaluation of cellular murine immune response in lymphocyte proliferation assay

Splenocytes collected from the mice immunized with the inactivated AMK-16 bacterial suspension were seeded at 1×10^5^ cells/mL into 96-well cell culture plates in DMEM/F12 (Biolot, Russia) supplemented with 10% fetal bovine serum, FBS (Sigma, USA) and 0.1% gentamicin (Sigma, USA). The cells were stimulated with the *C*. *psittaci* recombinant antigens, namely MOMP-AMK-16, DnaK-AMK-16, GrpE-AMK-16, MOMP-GR9, DnaK-GR9, and GrpE-GR9 (all in the dose of 10 μg/ml). In separate experiments, C-antigen was also used in similar concentration, 10 μg of protein/ml. The plates were incubated at 37 °C in a 5% CO_2_ atmosphere for 5 days. Unstimulated splenocytes and Concanavalin A from *Canavalia ensiformis* Type IV-S (ConA) (Sigma, USA) (2.5 μg/ml) were used as a negative and positive control, respectively. Lymphocyte proliferation was assessed with the use of Cell Proliferation ELISA BrdU chemiluminescent kit (Roche Applied Science, USA) according to the manufacturer’s protocol. Stimulation indexes (SI) were calculated as the mean relative light units per second (rlu/s) of the antigen-stimulated cells divided by the rlu/s of the negative control.

### Statistical analysis

The data from humoral and cellular immune response evaluation were analyzed and represented using the GraphPad Prism 6.01 (GraphPad software, San Diego, CA, USA). The Mann–Whitney test was used for detecting the differences between independent groups, and the Fisher’s exact test was applied in the case of dichotomous variable, with the p-value <0.05 considered as significant for both statistical methods.

## Results

### Brief whole genome characteristics of the *C*. *psittaci* AMK-16 strain

In this study, the initial whole genome sequencing which was performed using two platforms, NGS and MinION, successfully provided two separate contigs. The first of them corresponded to the *C*. *psittaci* bacterial circular chromosome of 1,152,497 bp and the other one was recognized as a single circular plasmid of 7,552 bp. As expected, similarly with other *C*. *psittaci* strains, the AMK-16 genome was relatively small and consisted of almost 1,000 coding sequences (CDSs), including all the three rRNAs types (5S, 16S and 23S), 38 tRNA genes, and 3 ncRNAs. In addition, 32 pseudo genes due to mainly frameshift mutations (n = 24 of 32) and the presence of stop codons in at least 9 of 32 DNA sequences (S3 Table in S1 File) were found. Importantly, the G+C content of the AMK-16 strain was 39.1%, which has been found to be identical to that for *C*. *psittaci* strains [23]. The plasmid pAMK had eight coding sequences as in other known plasmid-bearing strains of *Chlamydia* spp. [23, 59, 60].

**Fig 1:**
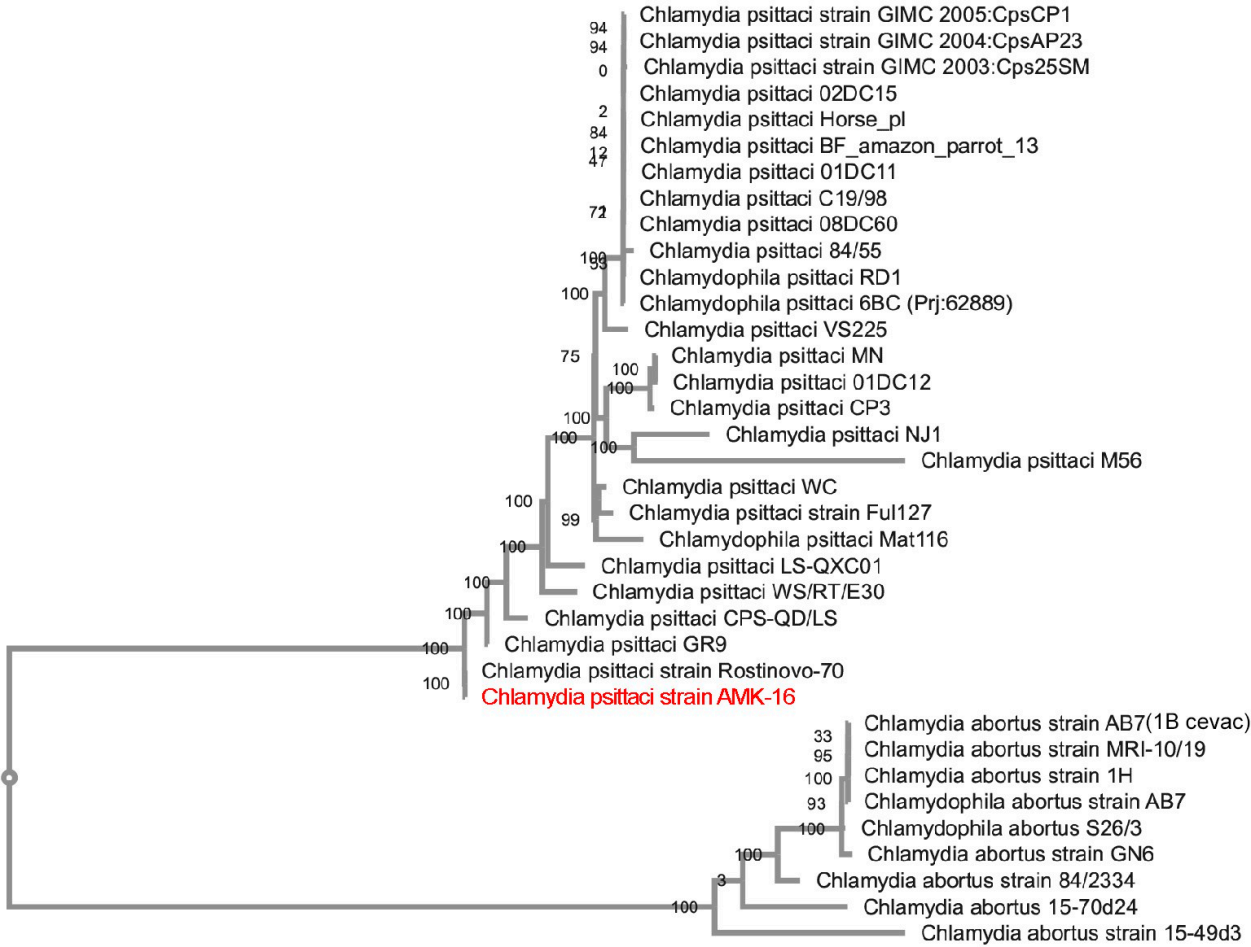
Phylogenetic tree based on the whole genome sequences of the *C*. *psittaci* AMK-16, the *C*. *psittaci* (n = 27) and the *C*. *abortus* strains (n = 9) isolated from different sources with the status of ‘complete assembly level’ available in the NCBI GenBank (https://www.ncbi.nlm.nih.gov/genbank/). The *C*. *psittaci* strain AMK-16 (marked in red) is clustered with only the *C*. *psittaci* Rostinovo-70 strain. The tree was generated on BV-BRC using Bacterial Genome Tree tool [52].

Phylogenetic analysis based on the whole genomes of all *C*. *psittaci* (n = 27) and *C*. *abortus* (n = 9) strains currently available in the NCBI GenBank database (https://www.ncbi.nlm.nih.gov/genbank/, accessed on 06 June 2023) and isolated from different sources demonstrated that the AMK-16 strain clustered only with *C*. *psittaci* but not with *C*. *abortus* strains (Fig 1). The AMK-16 formed a single branch only with another *C*. *psittaci* strain, also isolated from small ruminants [22], the *C*. *psittaci* Rostinovo-70 (Fig 1). The *C*. *psittaci* strain GR9, which was earlier (in 1960) isolated from a mallard in Germany [61], was identified as the closest homologue similarly with another study [62] and used as the reference one. Importantly, the *C*. *psittaci* strain 6BC, which was earlier recognized as the reference strain for the majority of *C*. *psittaci* representatives [63], formed another separate cluster of 15 relevant homologous strains. This cluster was phylogenetically distant from the other branch including, besides the AMK–16 & Rostinovo–70, and GR9, three other strains, the CPS-QD/LS, the WS/RT/E30 and the LS-QXC01, which, together with the AMK-16, formed a single cluster (Fig 1). The remaining *C*. *psittaci* strains formed three additional separate branches, each represented by a few (from 1 to 3) *C*. *psittaci* strains, such as: (i) the MN, the 01DC12 and the CP3; (ii) the NJ1, and (iii) the M56. The last two branches formed by two *C*. *psittaci* strains, the NJ1 and the M56, and isolated in the middle of the XX century, from either turkey or muskrat, were the most distant branches from the AMK-16-associated one (S1 Table in S1 File). These data could indicate the clear phylogenetic relation of the AMK-16 to *C*. *psittaci* species and divergence in relation to the most extensive cluster with the reference strain 6BC and to the minor ones as well.

Comparative analysis of the whole genomes of the *C*. *psittaci* AMK-16 versus the GR9 reference strain on the BV-BRC platform (https://www.bv-brc.org/) showed 100% homology in at least 865 CDSs, while 139 CDSs demonstrated a homology ranging from 73.4–99.9% (S4 Table in S1 File). Also, 41 CDSs were present in only the GR9 strain and absent in the AMK-16 strain. In fact, at least 35/41 CDSs were identified as uncharacterized hypothetical proteins. The remaining 6 CDSs might encode the following products: Phosphoglycolate phosphatase (EC 3.1.3.18), Acetyl-coenzyme A carboxyl transferase alpha chain (EC 6.4.1.2), Transcription-repair coupling factor, CHLTR possible phosphoprotein, Uncharacterized protein CT719 and DNA mismatch repair protein MutL.

**Fig 2:**
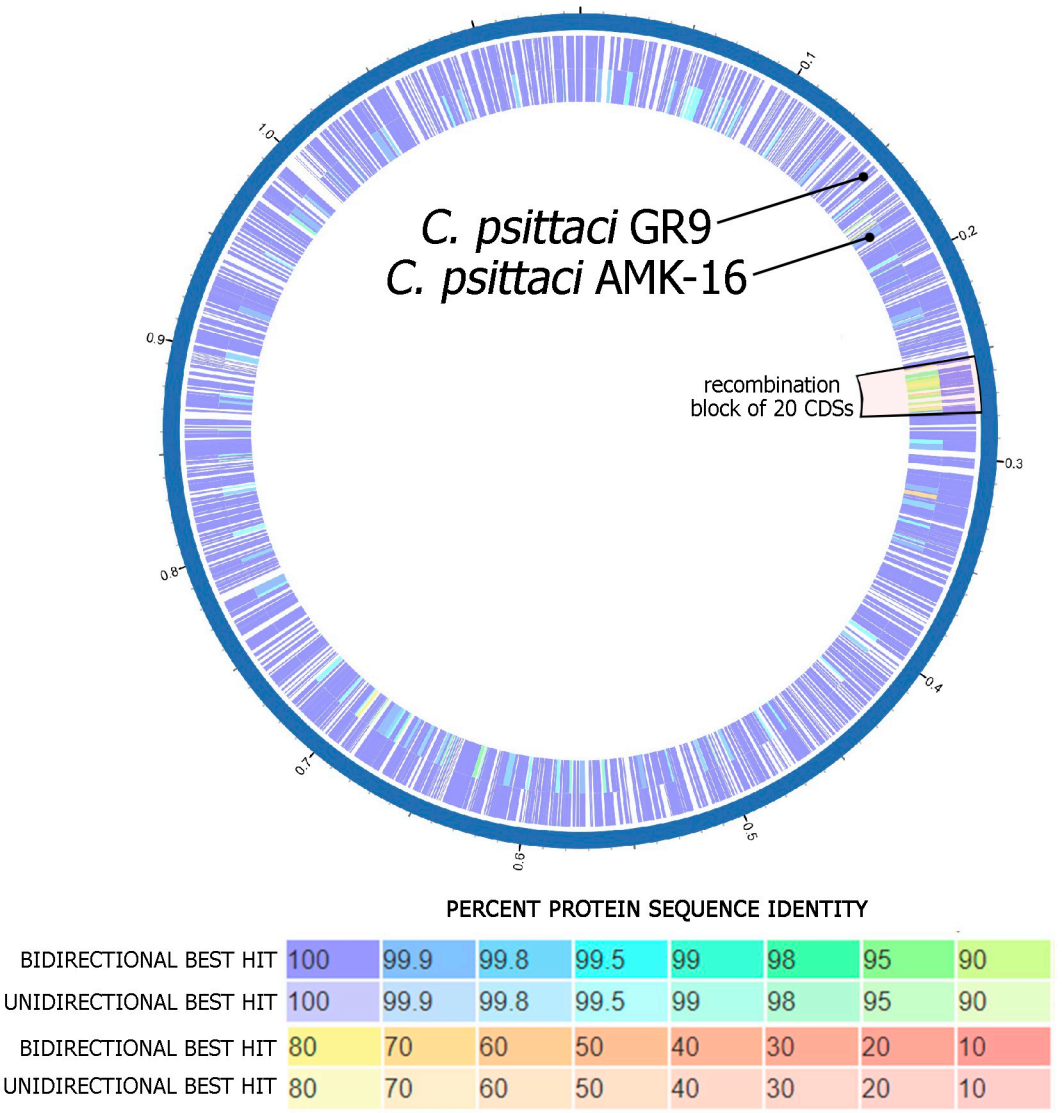
Circular representation of the genomes of the *C*. *psittaci* AMK-16 and the GR9 reference strain. The two circles inside (the purple diagram) show the distribution of the positions for protein-coding genes. The CDS identity is determined on a colorimetric scale, where purple/blue colors correspond to a higher percentage of identity than orange/red colored regions. The white areas represent the intergenic space in the chromosome sequence of the corresponding strain. The Figure was generated using the Proksee online tool (https://proksee.ca/, accessed on 06 June 2023).

Fig 2 highlighted a single chromosome region with a size of 23,100 b.p. located within 253 bp– 276 bp from the replication point of the AMK–16 chromosome. This region included the 20 CDSs together with intergenic spaces which were arranged along the AMK-16 chromosome as a separate block. The relevant genes demonstrated a relatively low homology (from 74.7 to 98.8%, on average– 91.9%) compared with those of the *C*. *psittaci* GR9 reference strain (S4 Table in S1 File).

As expected, in BLAST analysis (https://blast.ncbi.nlm.nih.gov/Blast.cgi), this region, following the removal of other AMK-16 genome regions, showed a similar level of low homology (92.2%) to the corresponding areas of the whole genome sequences derived from the majority of the *C*. *psittaci* strains (n = 27) available in the NCBI GenBank (https://www.ncbi.nlm.nih.gov/) independently from the isolation host (S5 Table in S1 File). A similarly low identity for this AMK-16 region (92.15% with 100% cover) was found with the analogous region in the *C*. *psittaci* GR9 reference strain. Only a single *C*. *psittaci* strain, the Rostinovo-70 [22], which had been earlier (more than 50 years ago) isolated from farm animals in Russia (S1 Table in S1 File), demonstrated the best match (100%) of the relevant region with that of the AMK-16 (S5 Table in S1 File). In fact, this AMK-16 region had a higher homology (in 98.38–99.94% range, with a total average of 98.57%) with the corresponding regions of all *C*. *abortus* strains (n = 11) (S5 Table in S1 File) which are currently also publicly available in the NCBI GenBank (https://www.ncbi.nlm.nih.gov/) and were isolated earlier from farm animals, humans, and birds worldwide. This AMK-16 region showed the highest homology (99.94%) with the relevant region of the *C*. *abortus* 15-70d24 isolated recently, in 2015, from the wild bird (*Anas crecca*) in Poland (S5 Table in S1 File).

**Fig 3:**
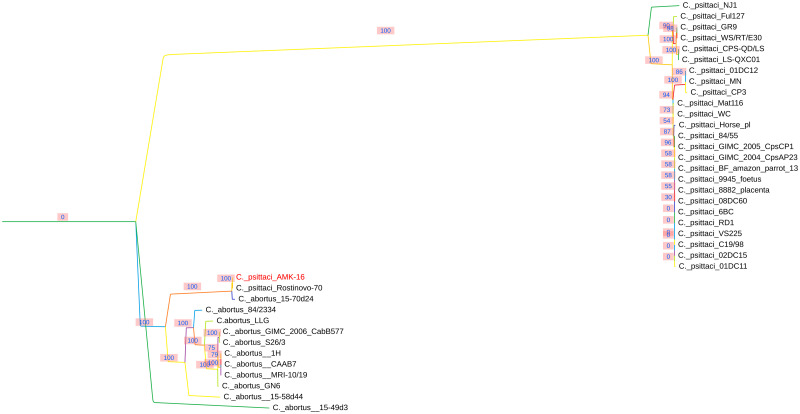
Phylogenetic tree based on the *C*. *psittaci* AMK-16 recombination block and the relevant regions found in the whole genome sequences of the *C*. *psittaci* strains (n = 25) available in the NCBI GenBank (https://www.ncbi.nlm.nih.gov/genbank/). This region of the *C*. *psittaci* AMK-16 is only clustered with the homologous regions of the *C*. *abortus* strains (n = 11) and the *C*. *psittaci* Rostinovo-70 genome [22], but not with the other representatives of *C*. *psittaci* indicated in S5 Table in S1 File.

Phylogenetic analysis of this *C*. *psittaci* AMK-16 region through the relevant regions of the *C*. *psittaci* and *C*. *abortus* strains available in the NCBI GenBank (https://www.ncbi.nlm.nih.gov/genbank/) (n = 36) proved the strict division into 2 separate clades or branches. The first of them was formed by the homologous sequences of only *C*. *psittaci* representatives, and the second one was combined exclusively by the *C*. *abortus* strains, including the AMK-16 (Fig 3).

These data could indicate a possible gene recombination event(s) in the relevant region of the AMK-16 similarly with the Rostinovo-70 [22] and other *Chlamydia spp*. [23–25, 54].

Comparative analysis of the plasticity zone (PZ), which has been recently identified in the AMK-16 and some other *C*. *psittaci* strains [62] revealed no difference between this organism and the GR9 (S6 Table in S1 File). Both strains possessed 10/16 genes presented in the *C*. *psittaci* 6BC in which the PZ has been defined as the segment flanked by genes *accB* (5’) and *guaB* (3’) [62]. The same six genes, encoding: (i) MAC/perforin family protein, (ii) GMP synthase [glutamine-hydrolyzing], amidotransferase subunit (EC 6.3.5.2)/GMP synthase [glutamine-hydrolyzing], ATP pyrophosphatase subunit (EC 6.3.5.2), (iii) Inosine-5’-monophosphate dehydrogenase (EC 1.1.1.205)/CBS domain, and (iv-vi) three hypothetical proteins were missing in both AMK-16 and the GR9 in contrast to the 6BC (S6 Table in S1 File). In agreement with the recent report [62], similar modifications in the relevant regions were found in four phylogenetically close strains, the Rostinovo-70, the WS/RT/E30, the CPS-QD/LS and the LS-QXC01, but not in the other three more distant strains, the Full127, the Mat116 and the WC (Fig 1, S6 Table in S1 File).

### Molecular typing of the *C*. *psittaci* AMK-16 strain

#### MLST

**Fig 4:**
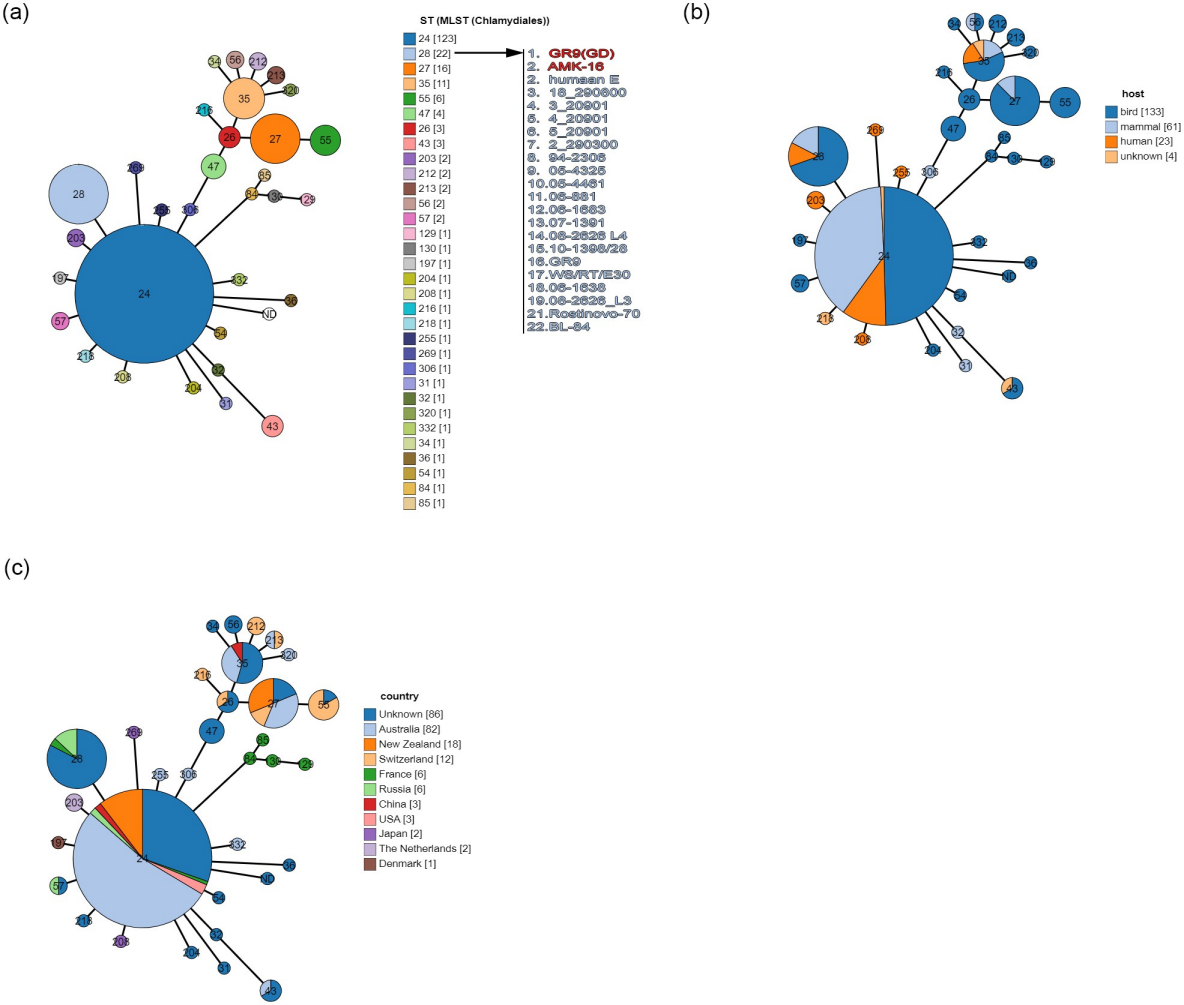
MLST Minimum spanning tree constructed using the GrapeTree algorithm based on the concatenated nucleotide sequences of 7 housekeeping genes (*gatA*, *oppA*, *hflX*, *gidA*, *enoA*, *hemN* and *fumC*) of the *C*. *psittaci* AMK-16 and all *C*. *psittaci* strains (n = 224) available in the PubMLST database (https://pubmlst.org/, accessed on 06 June 2023). The tree contains all sequence types (STs) currently identified in *C*. *psittaci* representatives. Each individual circle represents either: (a) a single sequence type (ST), or individual ST, sourced from specimens derived from: (b) different hosts, or (c) countries. The circle sizes are proportional to the number of strains. Links between circles represent the number of allelic mismatches between individual STs (S2 Table in S1 File). The AMK-16 belongs to ST28 presented as the light blue circle (a).

MLST analysis using the concatenated fragments of the seven *C*. *psittaci* housekeeping genes (*gatA*, *oppA*, *hflX*, *gidA*, *enoA*, *hemN* and *fumC*) [22, 23, 54, 55, 64] of the AMK-16 strain and all other *C*. *psittaci* strains (n = 224) available in the PubMLST database (https://pubmlst.org/) showed that the AMK-16 belonged to the ST28. It was the second largest cluster (n = 22), just after the ST24 (n = 123) (Fig 4a). Comparative analysis of the allele profiles of the AMK-16 and other representatives of ST28 and all the strains belonging to the ST24 demonstrated the difference in two variable alleles, *gatA* and *fumC* (S2 Table in S1 File).

The AMK-16 together with the Rostinovo-70 became the only strain of ST28 isolated from small ruminants (2/22, 9.1%). The majority of ST28 strains, including the GR9 and the WC/RT/E30 organisms, were initially derived from bird specimens, ducks, poultry, ibis and mallard (16/22, 72.7%), while fewer were sourced from mammals, either human (3/22, 13.6%) or fox (1/22, 4.6%) samples (S2 Table in S1 File, Fig 4b). In contrast, more diversity was revealed in ST24 group, which involved the strains from birds (different psittacine birds) and mammals (exclusively horses and humans). The AMK-16 belonged to ST28 strains, which were mainly distributed in the European part of Eurasia, France, and Russia, while there were a number of isolates with unknown sources (Fig 4c). The ST24 strains were more common in Australia and New Zealand, and occasionally in Russia, France, China, and the USA.

Based on MLST analysis, the AMK-16 as well as other ST28 strains were descendants only of the ST24 group, which founded a number of the first, second, third, fourth and fifth generations of descendants. In contrast, ST28 demonstrated the absence of currently detected descendants indicating potential evolution of the AMK-16 and, probably, other ST28 strains on some other *C*. *psittaci* genes.

#### *ompA*-typing

**Fig 5:**
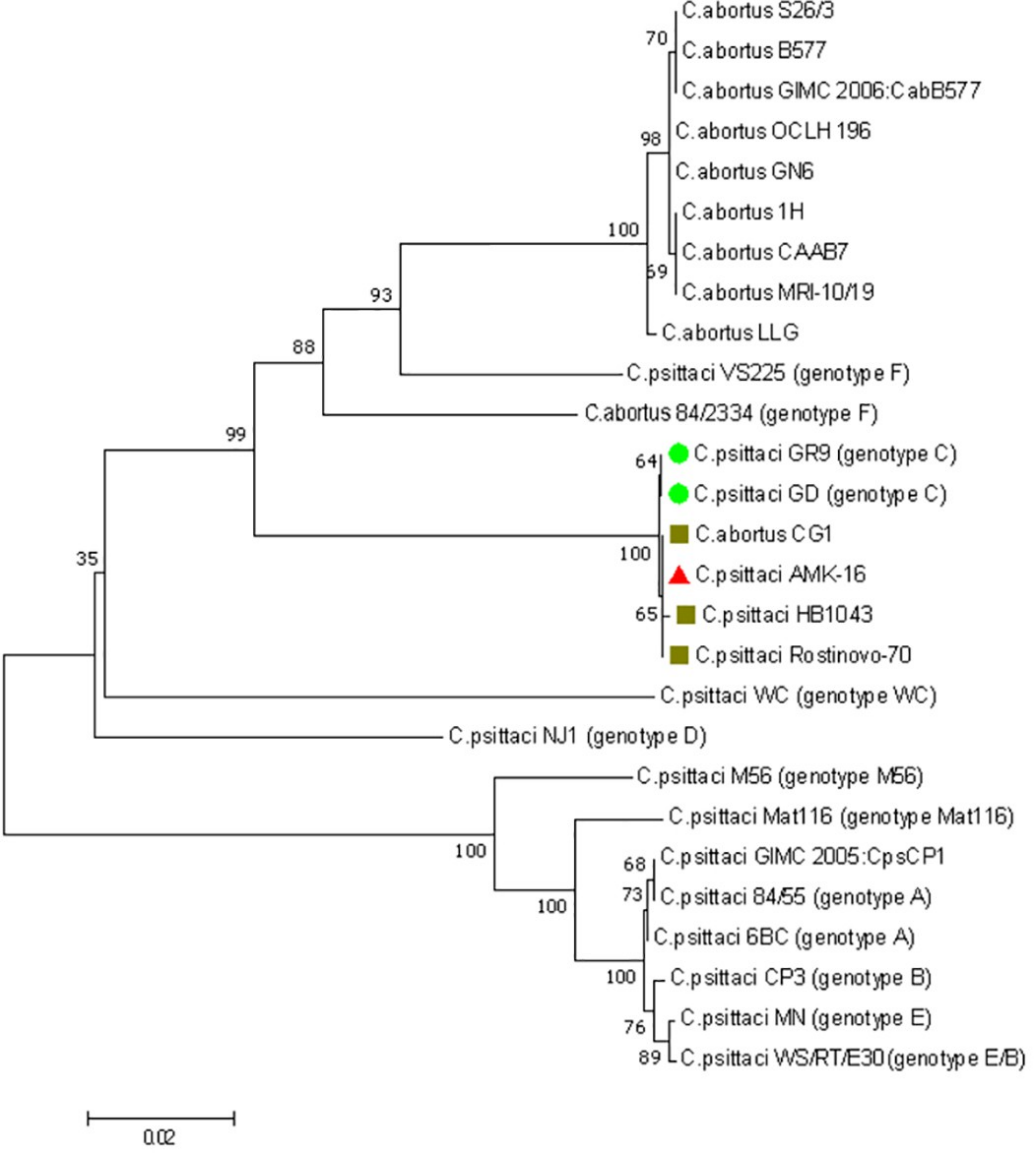
Phylogenetic tree based on the *ompA* gene sequences of the *C*. *psittaci* AMK-16 and the sequences of the *ompA* genes of the *C*. *psittaci* reference genotypes A—F, and *C*. *abortus* strains available in the NCBI GenBank (https://www.ncbi.nlm.nih.gov/genbank/). The *C*. *psittaci* strain AMK-16 is formed a single branch with only the *C*. *psittaci* Rostinovo-70 strain (four strains forming with the AMK-16 individual branch colored as: red triangle, the AMK-16; green circles, the GR9 and GC or dark green squares, the CG1, the HB1043 and the Rostinovo-70).

Phylogenetic analysis based on the *ompA* sequences derived from the ten *C*. *psittaci* classical genotypes (A—F, E/B, Mat116, M56 and WC) showed the marked difference with the AMK-16 sample. In fact, the AMK-16 differed from the representatives of all the ten *C*. *psittaci* identified genotypes, including the genotype C, and formed a single branch only with few chlamydia strains, the *C*. *psittaci* Rostinovo-70, the *C*. *psittaci* HB1043 (Acc. No. in the NCBI GenBank: JN411078.1) and the *C*. *abortus* CG1 (Acc. No. in the NCBI GenBank: EU531729) (Fig 5), which had also been isolated from farm animals. The AMK-16 was clearly different from two other *C*. *psittaci* strains of the same ST28, the GR9 of the genotype C and the WS/RT/E30 of the genotype E/B (S2 Table in S1 File, Fig 4a), which formed two other separate branches (Fig 5). Furthermore, based on the BLAST comparative analysis, the AMK-16 *ompA* sequence demonstrated either 100% or 99.9% identity with the relevant *ompA* sequences derived from either the *C*. *psittaci* Rostinovo-70 and *C*. *abortus* CG1 or the *C*. *psittaci* HB1043, respectively. In comparison with the *C*. *psittaci* GR9 reference strain of the genotype C, the nucleotide sequences derived from the *C*. *psittaci* AMK-16 and all the other three strains indicated, the *C*. *psittaci* Rostinovo-70, the *C*. *abortus* CG1 and the *C*. *psittaci* HB1043, demonstrated two identical single nucleotide polymorphisms (SNPs) in the positions 488 (G → A, G488A) and 984 (G → C, G984C) (S1a Fig in S1 File). Each of them resulted in a non-synonymous substitution of the relevant amino acid (N, Asn, Asparagine) → S (Ser, Serine) in the position 163 (N163S) and I (Ile, Isoleucine) → M (Met, Methionine) in the position 328 (I328M) (S1b Fig in S1 File). An additional single SNP in the position 1158 (T → C, T1158C) was detected in the *ompA* nucleotide sequence of the *C*. *psittaci* HB1043 strain as a possible silent mutation with no modification in the relevant amino acid, N → N (Asn → Asn, N386N) (S1a, S1b Fig in S1 File). In any case, the presence of this SNP could shed light on polymorphism followed by the appearance of an additional short branch inside the clade which was formed by all four strains, the *C*. *psittaci* AMK-16, the Rostinovo-70, the HB1043, and the *C*. *abortus* CG1 (Fig 5). Based on the marked difference of the *ompA* sequences derived from the AMK-16 and the other three homologous strains from that of the representatives of the *C*. *psittaci* genotype C, the GR9 and the GD, as well as other classical *C*. *psittaci* genotypes (Fig 5), the AMK-16 together with the Rostinovo-70 and the *C*. *abortus* CG1 could belong to either the novel *C*. *psittaci* genotype G (following through the well-known classical A to F) or the subtype C1. However, the first suggestion may be more feasible, as the AMK-16 together with the other three strains formed a distinct branch which was clearly separated from the branch formed by two strains, the GR9 and the GD, and not overlapping either of them (Fig 1). In this case, the *C*. *psittaci* HB1043 could be further easily subtyped as the G1 genotype. In fact, the *ompA* sequence of the *C*. *abortus* CG1 was identical to the relevant gene of the AMK-16 and the Rostinovo-70, the whole genomes of which have been clearly proven to belong to *C*. *psittaci* spp. Thus, the *ompA*-based genotyping indicated that the CG1 strain could be reclassified as the *C*. *psittaci* CG1. However, neither the whole genome nor some other molecular targets of this strain are currently available to make the final decision on this matter. Similarly to the AMK-16 and the three other strains, the Rostinovo-70, the NB1043 and the CG1, the *C*. *psittaci* strains of genotype A, the 6BC, the 84/55 and the GIMS 2005:CpsCP1 clustered separately from the genotypes B, E and E/B strains as a single/individual genotype (Fig 5). The *ompA* sequence of the GIMS 2005:CpsCP1 was identical to that of the 84/55, indicating the same genotype A for both strains. However, both strains demonstrated certain discrimination from the 6BC, which probably identified them as the genotype A1.

Nevertheless, the AMK-16 together with the other three strains, the Rostinovo-70, the HB1043 and, probably the CG1 clearly formed individual genetic lineage related to *ompA* polymorphism.

### Investigation of humoral and cellular immune responses induced by immunization with the inactivated *C*. *psittaci* AMK-16 strain in animal models

To evaluate the antibody response induced by the immunization of mice and ewes with the inactivated *C*. *psittaci* AMK-16 bacterial cells, an immunoblot with the panel of recombinant *C*. *psittaci* antigens, i.e., MOMPs, DnaK and GrpE, as well as with the MOMP-containing C-antigen and the *C*. *psittaci* AMK-16 bacterial lysate was performed. The comparison of nucleotide sequences for both DnaK and GrpE structural genes of the *C*. *psittaci* AMK-16 strain revealed a high (98.43–100% and 98.78–100%, respectively) level of identity with the relevant genes of *C*. *abortus* strains isolated from different animal hosts (S7 Table in S1 File). The identity of DnaK and GrpE nucleotide sequences for the AMK-16 and the GR9 strains was 98.8% and 95.3%, respectively (S4 Table in S1 File). However, the relevant genes, *dnaK* and *grpE*, demonstrated a marked polymorphism in the AMK-16 strain versus the reference GR9 as 60 SNPs and 38 SNPs, respectively (S8 Table in S1 File, S2a, S3a Figs in S1 File). These nucleotide modifications resulted in 7 and 9 nonsynonymous substitutions in the AMK-16 relevant amino acid sequences of DnaK and GrpE (S8 Table in S1 File, S2b, S3b Figs in S1 File).

All the mice (100%) immunized with the inactivated *C*. *psittaci* AMK-16 developed antibodies strictly specific to the MOMP AMK-16 (Table 1). Notably, these sera reacted not only with the MOMP of the homologous *C*. *psittaci* AMK-16 strain, but also with the MOMP of the wild type *C*. *psittaci* GR9 reference strain, indicating that the humoral response induced by the AMK-16-based immunization could be addressed against the infection caused by the wild type *C*. *psittaci* strains. Similarly, clear immunodominant specific reaction with the band of 42 kDa corresponding to the MOMP antigen was detected in 100% of murine antisera when both C-antigen and the AMK-16 lysate itself were fractionated and used as sensitines for immunoblotting. No reaction with the MOMP-containing antigens was observed with the sera from the control unimmunized group. Thus, the MOMP-directed humoral response was highly specific (p<0.001) for the group of mice immunized with the inactivated *C*. *psittaci* AMK-16 bacterial suspension (Table 1).

**Table 1:** Study of the humoral immune response induced by the immunization of mice with the inactivated *C*. *psittaci* AMK-16 bacterial suspension in immunoblotting with the panel of *C*. *psittaci* antigens.

*C*. *psittaci* antigens	Number of sera with positive reaction in groups of mice				P value ^2^
	Immunized with the inactivated *C*. *psittaci* AMK-16 bacteria (n = 6)		Control unimmunized (n = 7)		
	Abs.	%	Abs.	%	
**MOMP AMK-16**	6/6	100	0/7	0	<0.001
**MOMP GR9**	6/6	100	0/7	0	
**C-antigen AMK-16** ^1^	6/6	100	0/7	0	
**DnaK AMK-16**	4/7	57.1	3/6	50	ns
**DnaK GR9**	4/7	57.1	3/6	50	
**GrpE AMK-16**	2/7	28.6	2/6	33.3	
**GrpE GR9**	1/7	14.3	2/6	33.3	
***C*. *psittaci* AMK-16 lysate** ^1^	6/6	100	0/7	0	<0.001

^1^ The result was assigned as positive if the reactivity with the band of 42 kDa corresponding to the MOMP antigen was detected.

^2^ Fisher’s exact test was performed to compare the differences between groups.

Furthermore, slightly more than a half and less than a third of murine sera from the experimental group were positive to DnaK and GrpE proteins, respectively. This antibody response was likely non-specific, since the similar rate of positive responses was registered in the control group (p>0.05). Moreover, no significant differences have been found between the responses directed at these proteins of the *C*. *psittaci* homologous (the AMK-16) and wild-type (the GR9) strains (Table 1), despite a marked polymorphism in the AMK-16 genes encoding the AMK-16 DnaK and GrpE (S9 Table in S1 File).

As was expected, the dominant MOMP-directed humoral response was detected in the ewes vaccinated with the inactivated AMK-16 strain (S9 Table in S1 File). However, antibodies to both DnaK AMK-16 and DnaK GR9 were also registered in 87.5% and 62.5% of the immunized ewes, respectively. Only 25% of the ewes were seropositive to GrpE antigens.

**Fig 6:**
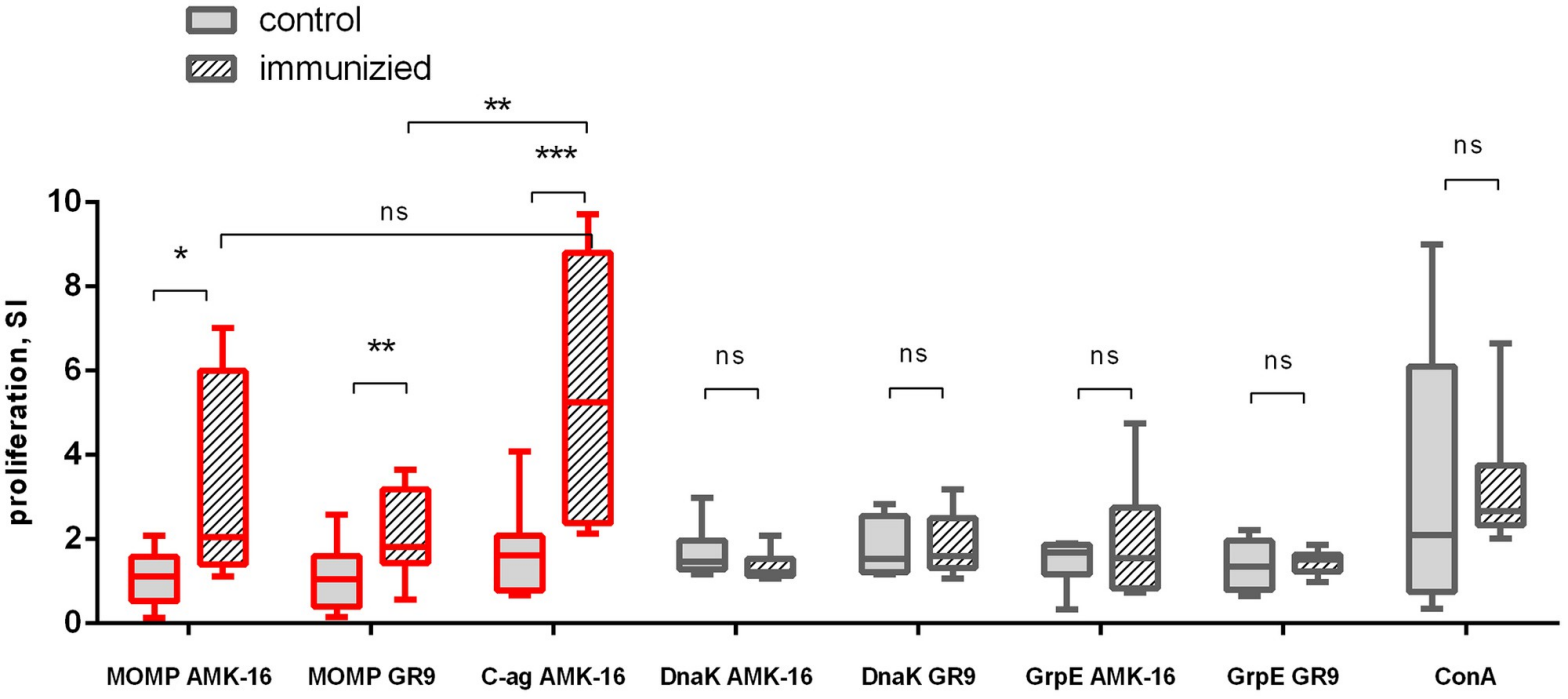
Proliferative cellular response to the panel of *C*. *psittaci* antigens in mice immunized with the inactivated *C*. *psittaci* AMK-16 strain and the control unimmunized animals. Splenocytes were stimulated with the *C*. *psittaci* antigens of the homologous AMK-16 strain (MOMP AMK-16, C-antigen AMK-16, C-ag, DnaK AMK-16 and GrpE AMK-16) and the wild type GR9 strain (MOMP GR9, DnaK GR9 and GrpE GR9), and stimulation indexes, SI, were calculated against the non-stimulated cells. The Mann Whitney test was performed for statistical analysis. Significantly differed cellular responses are designated as red boxes; ***—p<0.001, **—p<0.01, *—p<0.05, ns–not significant.

To assess the cellular antigen-specific murine immune response induced by the immunization with the inactivated *C*. *psittaci* AMK-16 bacterial suspension, a lymphocyte proliferation assay was performed. Middle lymphocyte proliferation was detected in the immunized group to both recombinant MOMP AMK-16 and MOMP GR9 stimuli (Fig 6). However, this response was significantly higher than those detected in the control unimmunized mice (p<0.05 and p<0.01, respectively). For the immunized group, the highest stimulation indexes, SI, were obtained to the C-antigen stimulus (p<0.001). This could indicate that the specific cellular immune response was directed not only at the MOMP, but also at the other components of the C-antigen, known as the ornithosis antigen, which is used for the conventional compliment fixation test in the investigation of EAE in ewes or chlamydia common antigen [39, 65]. No significant differences were observed in the lymphocyte proliferation activity induced by ConA between the groups, as well as in the proliferative response directed at both DnaK and GrpE proteins either of the homologous (the AMK-16) or the wild type (the GR9) *C*. *psittaci* strains, although a marked polymorphism of both AMK-16 antigens from the GR9 DnaK and the GR9 GrpE was detected (S8 Table in S1 File, S2b, S3b Figs in S1 File).

## Discussion

In this study, the AMK-16 strain that was recently isolated from the caprine aborted fetal tissue was investigated. This strain has demonstrated virulence in mouse model causing the death of 20, 70 and 100% of mice after subcutaneous, intraperitoneal, or intranasal infection, respectively. Moreover, the AMK-16 was virulent for guinea pigs [37]. Simultaneously, the inactivated suspension of the AMK-16 strain showed a good level of protection against experimental chlamydia infection caused by *C*. *psittaci* wild strains sourced from cattle or small ruminants in an animal model. In fact, the immunization of white mice with the inactivated suspension of the AMK-16 by *i*.*p*. route could result in 2–4.6-fold and more survived animals (up to 85%) compared with the unimmunized naïve control group (no more than 20–35% of survivors) after *i*.*p*. administration with either bovine or ovine, or porcine, or homologous caprine virulent wild *C*. *psittaci* strains [37]. Moreover, all 100% pregnant rabbits treated with the AMK-16 suspension following model infection with virulent *C*. *psittaci* wild strains, produced only alive offspring [37]. The presence of the AMK-16 specific serum antibodies detectable in the CFT of 100% of vaccinated animals certainly correlated with the protective immune response induced by the immunization with the AMK-16 [37]. However, the role of individual proteins in the elicited antibody or cellular immune responses has not been elucidated. Therefore, it was important to investigate the main molecular and immunological characteristics of this strain demonstrating its basic specific activities as a promising vaccine candidate for KWCV through whole genome sequencing followed by phylogenetic analysis, molecular typing, and comparison with the genomes of both C. *psittaci* and *C*. *abortus* strains available, and study of the immunodominant antigen target(s) of the AMK-16.

It is known that all *Chlamydia* possess a chromosome of the size around 1 Mbp which may be supplemented with an extrachromosomal circular replicon or cryptic plasmid of around 7.0–7.5 kbp in approximately 50–60% of isolated strains [23, 59, 60, 66–69]. The AMK-16 whole genome sequenced had a chromosome of about 1.2 Mbp and the cryptic plasmid of about 7.5 kbp (S3 Table in S1 File), which corresponded to typical chlamydia representative genomes [3, 22–24, 30, 59–63, 70]. Phylogenetic analysis based on the whole genome sequences of the AMK-16, *C*. *psittaci* and *C*. *abortus* strains available clear clustered the AMK-16 with only *C*. *psittaci* representatives (Fig 1). Furthermore, the AMK-16 had the GC content typical for *C*. *psittaci* [23] and belonged to ST28 which includes a number of *C*. *psittaci* strains (Fig 4a). Overall, this clustering was in agreement with the MLST clustering reported earlier [54, 62, 64, 70]. However, the AMK-16 became the second strain after the Rostinovo-70 grouped with the *C*. *psittaci* group II, which had initially included mainly the duck strains [54, 64]. Among them there was the *C*. *psittaci* GR9, the whole genome of which was found to be phylogenetically the closest homologue to the AMK-16 (Fig 1) similarly to the Rostinovo-70 as we reported earlier [22], which was then confirmed by recent studies from research groups [62, 64, 70]. Comparative analysis of the whole genomes of these two strains resulted in the detection of a possible recombination event in the AMK-16 chromosome region with the size of 23.1 kbp coding 20 CDSs (Fig 2). It was not surprising, because the cases of chlamydia infection in farm animals caused by Chlamydia strains with chromosomes affected by recombinations have been reported [22–25, 54]. Importantly, those recombination events have been identified in *C*. *psittaci* in contrast to the more stable *C*. *abortus* with either little or no homologous recombinations associated with subsequent genome modification [24]. As expected, this AMK-16 region was actually identical to that in *C*. *abortus* demonstrating a marked difference from those in *C*. *psittaci* representatives (S4, S5 Tables in S1 File). Recombination analysis based on the MaxChi method [53] strictly confirmed the phylogenetic position of this AMK-16 region in only the *C*. *abortus*-based cluster but not in the *C*. *psittaci*-related group (Fig 3). However, the comparison of the whole genomes of only the GR9 and the AMK-16 strains provided no marked difference in the PZ. That was revealed only when the 6BC and other *C*. *psittaci* strains, those phylogenetically more distant from the AMK-16 than the GR9 were compared (Fig 1, S6 Table in S1 File). In fact, at least six genes were lost in this region of the AMK-16. It is not clear whether or not some of them could be involved into the protective immune response conferring by the immunization of animal models with the AMK-16. These findings agree with the recent research, in which PZ has been carefully investigated in a number of *C*. *psittaci* strains [62].

A panel of recombinant or chemically derived proteins of the AMK-16 and the reference GR9 strains was used to evaluate both humoral and cellular immune responses elicited by the inactivated *C*. *psittaci* AMK-16 strain. The panel included the well-known immunodominant chlamydia antigen MOMP, MOMP-containing C-antigen and two proteins, Chaperone protein DnaK and Heat shock protein GrpE, encoded within the potential recombinational region of the *C*. *psittaci* AMK-16 strain. DnaK and GrpE are the stress response proteins, whose immunogenic and potentially protective properties were recently shown in different bacterial models [46, 47, 71, 72]. Both proteins demonstrated a certain variability in the AMK-16 compared with the GR9 (S2, S3 Figs in S1 File), while having almost identical amino acid lengths, 658/659 (DnaK) and 191/191 (GrpE), respectively (S4 Table in S1 File). For this reason, it was intriguing whether DnaK and GrpE could participate in the specific immune response induced by the immunization with the inactivated *C*. *psittaci* AMK-16 bacterial cells. In fact, at least one of these proteins, DnaK, clearly participated in the pathogen-host interaction following recognition by the host immune system of animal models, such as mice and ewes. Thus, DnaK could certainly support only the humoral host immune response in half of the mice immunized with the inactivated AMK-16 bacteria cells as the experimental model chlamydial KWCV (Table 1). In contrast, this protein showed better immunoreactivity in the target animal species, ewes, the sera of the majority of which tested positive for the presence of anti-DnaK antibodies (S7 Table in S1 File). GrpE clearly demonstrated negligible participation in the AMK-16-mediated antibody and cellular responses in only single animals in both mouse and ovine models (Fig 6).

However, two other AMK-16 antigens, MOMP and MOMP-containing C-antigen, were identified as the AMK-16 immunodominant proteins, while demonstrating less polymorphism than both the AMK-16 DnaK and GrpE (S8 Table in S1 File, S2, S3 Figs in S1 File). Both humoral and cellular responses elicited by immunization with the inactivated *C*. *psittaci* AMK-16 strain were strictly MOMP-specific. Notably, these responses were targeted not only at the MOMP of the homologous *C*. *psittaci* AMK-16 strain, but also at the MOMP of wild type *C*. *psittaci* GR9. These findings are in good accordance with previous reports which state that the immune response induced by immunization with the inactivated *C*. *psittaci* AMK-16 could be effective against the infection caused by the wild type *C*. *psittaci* strains of different animal sources. Further on, the recombinant MOMP derived from the AMK-16 induced more marked T-cell proliferative response than that from the GR9 strain (Fig 6), although it was not statistically significant in a mouse model (p<0.05). In any case, this finding could indicate the importance for the protective host immune response of two non-synonymous substitutions in the nucleotide sequence of the *ompA* gene encoded MOMP in the *C*. *psittaci* strains with the novel genotype G (S1 Fig in S1 File), which were already detected in some countries, such as Russia and China. Importantly, this protein, MOMP, possessing both B- and T-cell immunoreactive epitopes, demonstrated high immunogenic activity. Therefore, nowadays natural and recombinant MOMP has been recognized as the most promising antigen for chlamydia vaccine development which completed the human clinical trial of Phase I [40–44, 73–75]. The data obtained in the current study could indicate that: (i) MOMP and DnaK were the two immunodominant antigens eliciting the host immune response in both laboratory and target animals after their immunization with the inactivated AMK-16 bacteria; (ii) the AMK-16 may be a promising vaccine candidate for development of veterinary KWCV against chlamydia infection, including EAO, in ruminants. In fact, the marked protective activity of the AMK-16 could be related to the specific genome characteristics, such as: (i) polymorphism in the *ompA* gene, resulting in the modification in MOMP amino acid compositions, which could lead to further evolution of *C*. *psittaci* strains and formation of the *C*. *psittaci* novel genotype variant that was differed from the genotype C; (ii) the presence in the *C*. *psittaci* chromosome a recombinant region highly homologous to *C*. *abortus* which encoded protein(s) with marked immunogenic potency.

Some limitations of our study should be acknowledged. The main limitation was connected with the availability of a certainly small number of *C*. *psittaci* whole genomes with the status of “complete assembly level” which could be used to study the recombination event in these microorganisms. To avoid any inaccuracy in the phylogenetic relationships of the AMK-16 with other chlamydia, the extended collection of *C*. *psittaci* strains (n = 224) was carefully investigated using the PubMLST tools. Furthermore, the whole genomes of only a few *C*. *psittaci* strains, the Rostinovo-70 and the AMK-16 isolated in Russia, have been carefully investigated so far. Although both belonged to a single well-known clonal lineage (ST28) with other *C*. *psittaci* strains detected worldwide, they demonstrated a certain intraspecific genomic variation compared with others since the novel genotype G has been revealed. However, more isolates are needed to unravel the biodiversity of *C*. *psittaci* strains and the frequency of occurrence of strains with this genotype in different countries including Russia. Secondly, the present study was exclusively concentrated on the antigen-specific immune response elicited by the immunization with the inactivated AMK-16 bacteria, not their protective efficacy, since the high protective potential of the inactivated AMK-16 has been recently demonstrated in different laboratory models [37]. Further, we did not measure the antibody titers in ELISA, only assessing antigen-antibody specific interaction in immunoblot assay to discover immunoreactive antigens of the *C*. *psittaci* AMK-16 strain, including those encoded within the recombinational insertion from *C*. *abortus*. The next limitation was that the panel of antigens used for serological and cellular response studies included only the AMK-16- and the GR9-derived antigens lacking those from other *C*. *psittaci* as well as from *C*. *abortus* strains, which would be advisable to test cross-reactivity with these two common pathogens of farm animals. Finally, although we assessed the proliferative cellular response induced by the inactivated AMK-16, we did not yet evaluate the antigen-specific cytokine profiles in both mice and target animals, although it was shown that protective immunity against chlamydia infection is strongly associated with the Th1-polarized immune response [76–80].

## Conclusion

Our findings could result in an enhanced host immune response in animals immunized with the AMK-16. The KWCV based on the inactivated AMK-16 could be safe with no live vaccine-related complications in the form of clinical manifestations similar to chlamydia disease. Moreover, this vaccine may be universally protective for small ruminants and other livestock against *C*. *psittaci* strains sourced from different animal species, such as sheep, goats, pigs and cattle, as was reported recently [24, 38]. This vaccine, through the presence in the formulation of antigen composition from both *C*. *psittaci* and *C*. *abortus*, has a potency to protect animals infected with chlamydia belonging to either one of these pathogens or both of them. In fact, this characteristic needs to be carefully investigated in our future research.

## Supporting information

S1 File (ZIP)
