# Supplementary material for: Whole genome sequencing characteristics of Chlamydia psittaci caprine AMK-16 strain, a promising killed whole cell veterinary vaccine candidate against chlamydia infection
Source: PLoS One. 2023 Oct 30;18(10):e0293612. doi: 10.1371/journal.pone.0293612 (PMC10615304; doi:10.1371/journal.pone.0293612)
Supplement: S1 File — (ZIP) [file pone.0293612.s001.zip › Feodorova_S3_S5_S7_S8_S9 Tables.doc]

**S3 Table. Brief characteristics of the *C. psittaci* strain AMK-16 complete genome after the automatic genome annotation that was generated based on the NCBI Prokaryotic Genome Annotation Pipeline (PGAP) (https://www.ncbi.nlm.nih.gov/genome/annotation_prok/).**

| **Number of genes** | | | | | | | | | | | | **Number of Pseudo Genes** | | | | | | **GC, %** |
| --- | --- | --- | --- | --- | --- | --- | --- | --- | --- | --- | --- | --- | --- | --- | --- | --- | --- | --- |
| **Totally** | **CDSs** | **Coding proteins** | **Genes (RNA)** | **rRNAs** | | | **complete rRNAs** | | | **tRNAs** | **ncRNAs** | **Totally** | **CDSs (without protein)** | **Ambiguous residues** | **Frame-**  **shifted** | **Incomplete** | **With internal stop** |  |
|  |  |  |  | **5S** | **16S** | **23S** | **5S** | **16S** | **23S** |  |  |  |  |  |  |  |  |  |
| 1,025 | 981 | 949 | 44 | 1 | 1 | 1 | 1 | 1 | 1 | 38 | 3 | 32 | 32 | 0 of 32 | 24 of 32 | 5 of 32 | 9 of 32 | 39,12 |

**S5 Table. Comparative homology of the AMK-16 region with possible recombination event with the relevant regions in other *Chlamydia* strains in BLAST analysis (https://blast.ncbi.nlm.nih.gov/Blast.cgi).**

| **No.** | **Strain ID** | **NCBI GenBank Accession** | **Query Cover, %** | **Percent of Identity** | **Host** | **Source** |
| --- | --- | --- | --- | --- | --- | --- |
| 1 | *C. psittaci* Rostinovo-70 | CP041038.1 | 100 | 100.0 | Sheep | [1,2] |
| 2 | *C. abortus* 15-70d24 | LS450958.2 | 100 | 99.94 | *Anas crecca* | [3] |
| 3 | *C. abortus* 84/2334 | CP031646.1 | 100 | 98.65 | Parrot | [3,4-7] |
| 4 | *C. abortus* LLG | CP018296.1 | 100 | 98.51 | Goat | [3,8-11] |
| 5 | *C. abortus*  GIMC 2006:CabB577 | CP024084.1 | 100 | 98.42 | Sheep | N/A^1^ |
| 6 | *C. abortus* GN6 | CP021996.1 | 100 | 98.42 | Yak | [1,9,12] |
| 7 | *C. abortus* S26/3 | CR848038.1 | 100 | 98.42 | *Ovis aries* | [3,9,13] |
| 8 | *C. abortus* 1H | LN554883.1 | 100 | 98.40 | N/A | [14-17] |
| 9 | *C. abortus* CAAB7 | LN554882.1 | 100 | 98.40 | N/A | N/A |
| 10 | *C. abortus* MRI-10/19 | CP070224.1 | 100 | 98.40 | Sheep | [3] |
| 11 | *C. abortus* 15-58d44 | LS974600.1 | 99 | 98.39 | *Pica pica* | [3] |
| 12 | *C. abortus* 15-58d44 | OU508367.1 | 100 | 98.38 |  |  |
| 13 | *C. psittaci* WC | CP003796.1 | 100 | 92.20 | Cow | [1,3,18,19] |
| 14 | *C. psittaci* GIMC 2005:CpsCP1 | CP024451.1 | 100 | 92.19 | Human | [1,23] |
| 15 | *C. psittaci* GIMC 2004:CpsAP23 | CP024455.1 | 100 | 92.19 |  | [1,23] |
| 16 | *C. psittaci* BF_amazon_parrot_13 | CP110211.1 | 100 | 92.19 | *Amazona aestiva* | [24] |
| 17 | *C. psittaci* 9945_foetus | CP092199.1 | 100 | 92.19 | *Equus caballus* | [27] |
| 18 | *C. psittaci* 8882_placenta | CP092197.1 | 100 | 92.19 |  |  |
| 19 | *C. psittaci* 08DC60 | CP002807.1 | 100 | 92.19 | Human | [26] |
| 20 | *C. psittaci* 6BC | CP002586.1 | 100 | 92.19 | Parakeet | [1,3,20,21,31,32] |
| 21 | *C. psittaci* 6BC | CP002549.1 | 100 | 92.19 |  |  |
| 22 | *C. psittaci* RD1 | FQ482149.1 | 100 | 92.19 | N/A | [1,22] |
| 23 | *C. psittaci* Horse_pl | CP025423.1 | 100 | 92.19 | *Equus caballus* | [25] |
| 24 | *C. psittaci* C19/98 | CP002804.1 | 100 | 92.19 | *Ovis aries* | [26] |
| 25 | *C. psittaci* 02DC15 | CP002806.1 | 100 | 92.19 | Cattle | [26] |
| 26 | *C. psittaci* 01DC11 | CP002805.1 | 100 | 92.18 | Pig | [26] |
| 27 | *C. psittaci* Ful127 | CP033059.1 | 100 | 92.18 | Fulmars | [1,28] |
| 28 | *C. psittaci* VS225 | CP003793.1 | 100 | 92.17 | *Aratinga canicularis* | [1,3,18,19] |
| 29 | *C. psittaci* 84/55 | CP003790.1 | 100 | 92.17 | Parakeet |  |
| 30 | *C. psittaci* GR9 | CP003791.1 | 100 | 92.15 | *Anas platyrhynchos* (Duck) |  |
| 31 | *C. psittaci* WS/RT/E30 | CP003794.1 | 100 | 92.14 | *Anas platyrhynchos* |  |
| 32 | *C. psittaci* CPS-QD/LS | CP103952.1 | 100 | 92.14 | Duck | N/A |
| 33 | *C. psittaci* Mat116 | CP002744.1 | 100 | 92.15 | *Ara severa* | [1] |
| 34 | *C. psittaci* LS-QXC01 | CP098512.1 | 100 | 92.12 | Human | N/A |
| 35 | *C. psittaci* NJ1 | CP003798.1 | 100 | 92.10 | *Meleagris gallopavo* | [1,3,7,19] |
| 36 | *C. psittaci* 01DC12 | HF545614.1 | 100 | 92.09 | Human | [3,27,29,30] |
| 37 | *C. psittaci* MN | CP003792.1 | 100 | 92.08 |  |  |
| 38 | *C. psittaci* CP3 | CP003797.1 | 100 | 92.06 |  | [1,3,7,19] |
| 39 | *C. psittaci* GIMC 2003:Cps25SM | CP024453.1 | 99 | 92.37 | Human | [1,23] |
| 40 | *C. psittaci* M56 | CP003795.1 | 99 | 92.55 | *Ondatra zibethicus* | [1,18,19] |

^1^ Not available (N/A).

**S7 Table**. **Analysis of nucleotide sequences identity between DnaK and GrpE structural genes of the *C. psittaci* AMK-16 and their orthologs**

**from *C. abortus* and *C. psittaci* strains.**

| **Gene** | **locus_tag** | **Protein** | **Homology with other chlamydia spp./strains** | **Query Cover,**  **%** | **Percent**  **of Identity** | **Host** |
| --- | --- | --- | --- | --- | --- | --- |
| *dnaK* | GR632_03940 | molecular chaperone DnaK | *C. psittaci* Rostinovo-70 | 100 | 100 | Ewe |
|  |  |  | *C. abortus* 15-70d24 | 100 | 100 | *Anas crecca* |
|  |  |  | *C. abortus* 84/2334 | 100 | 99.39 | Parrot |
|  |  |  | *C. abortus* GN6 | 100 | 99.29 | Yak |
|  |  |  | *C. abortus* LLG | 100 | 99.29 | Goat |
|  |  |  | *C. abortus* 1H | 100 | 99.29 | mutant derived from sheep |
|  |  |  | *C. abortus* CAAB7 | 100 | 99.29 | N/A |
|  |  |  | *C. abortus* S26/3 | 100 | 99.24 | *Ovis aries* |
|  |  |  | *C. abortus* 15-49d3 | 100 | 98.43 | *Anas platyrhynchos* |
| *grpE* | GR632_03945 | nucleotide exchange factor GrpE | *C. psittaci* Rostinovo-70 | 100 | 100 | Ewe |
|  |  |  | *C. abortus* 15-70d24 | 100 | 100 | *Anas crecca* |
|  |  |  | *C. abortus* 84/2334 | 100 | 98.96 | Parrot |
|  |  |  | *C. abortus* 15-58d44 | 100 | 98.96 | *Pica pica* |
|  |  |  | *C. abortus* GIMC 2006:CabB577 | 100 | 98.78 | Sheep |
|  |  |  | *C. abortus* strain GN6 | 100 | 98.78 | Yak |
|  |  |  | *C. abortus* LLG | 100 | 98.78 | Goat |
|  |  |  | *C. abortus* 1H | 100 | 98.78 | mutant derived from sheep |
|  |  |  | *C. abortus* CAAB7 | 100 | 98.78 | N/A |
|  |  |  | *C. abortus* strain S26/3 | 100 | 98.78 | *Ovis aries* |
|  |  |  | *C. abortus* 15-49d3 | 100 | 96.8 |  |

**^S8 Table. Polymorphism in the AMK-16^ *^dnaK^* ^and^ *^grpE^* ^genes resulted in nonsynonymous amino acid substitutions.1^**

| **Product** | **No.** | ***C. psittaci* GR9** | |  | ***C. psittaci* AMK-16** | |
| --- | --- | --- | --- | --- | --- | --- |
|  |  | **Nucleotide sequence composition** | **AA sequence composition*** | **Substitutions in the nucleotide sequence** | **AA Substitutions in the protein** | **nonsynonymous substitution** |
| DnaK | 1 | GCA | Ala/A | TCA | Ser/S | A98S |
|  | 2 | ATG | Met/M | ATA | Ile/I | M124I |
|  | 3 | AGC | Ser/S | AAC | Asn/N | S309N |
|  | 4 | GAA | Glu/E | GAT | Asp/D | E508D |
|  | 5 | AAC | Asn/N | AGC | Ser/S | N549S |
|  | 6 | ACG | Thr/T | TCA | Ser/S | T572S |
|  | 7 | ACA | Thr/T | GCT | Ala/A | T641A |
| GrpE | 1 | GAA | Glu/E | GCA | Ala/A | E7A |
|  | 2 | TCC | Ser/S | CCA | Pro/P | S13P |
|  | 3 | AAA | Lys/K | AAC | Asn/N | K118N |
|  | 4 | TTC | Phe/F | GTC | Val/V | F146V |
|  | 5 | GTC | Val/V | ATC | Ile/I | V151I |
|  | 6 | GCA | Ala/A | GTA | Val/V | A176V |
|  | 7 | ACA | Thr/T | GCA | Ala/A | T178A |
|  | 8 | AAA | Lys/K | ACA | Thr/T | K182T |
|  | 9 | GAA | Glu/E | GCA | Ala/A | E184A |

^1^ The relevant GR9 *dnaK* and *grpE* genes and the gene products were used as the references.

**S9 Table.** **Study of the humoral immune response induced by an immunization of ewes with the inactivated *C. psittaci* AMK-16 bacterial suspension**

**in immunoblotting with the panel of *C. psittaci* antigens.**

| ***C. psittaci* antigens** | **Number of sera with positive reaction in groups of ewes (n = 8)** | |
| --- | --- | --- |
|  | Abs. | % |
| MOMP AMK-16 | 8/8 | 100 |
| MOMP GR9 | 8/8 | 100 |
| C-antigen AMK-16 ^1^ | 8/8 | 100 |
| DnaK AMK-16 | 7/8 | 87,5 |
| DnaK GR9 | 5/8 | 62,5 |
| GrpE AMK-16 | 2/8 | 25 |
| GrpE GR9 | 2/8 | 25 |
| *C. psittaci* AMK-16 lysate ^1^ | 8/8 | 100 |

^1^ The result was assigned as positive if the reactivity with the band of 42 kDa corresponding to the MOMP antigen was detected.

^2^ Fisher’s exact test was performed to compare differences between groups.

(**a**)

(**b**)

**S1 Fig. Multiple sequence alignment of the nucleotide (a) and amino acid (b) sequences of the *ompA* gene derived from the *C. psittaci* AMK-16, *C. psittaci* GR9, *C. psittaci* Rostinovo-70, *C. abortus* CG1 and *C. psittaci* HB1043 with the use of the online service MultAlin (http://multalin.toulouse.inra.fr/multalin/).** The SNPs are colored in blue.

**(a)**

(**b**)

**S2 Fig. Multiple sequence alignment of the nucleotide (a) and amino acid (b) sequences of the *dnaK* gene derived from the *C. psittaci* AMK-16 and *C. psittaci* GR9 with the use of the online service MultAlin (http://multalin.toulouse.inra.fr/multalin/).** The SNPs are colored in blue.

**(a)**

(**b**)

**S3 Fig. Multiple sequence alignment of the nucleotide (a) and amino acid (b) sequences of the *grpE* gene derived from the *C. psittaci* AMK-16 and *C. psittaci* GR9 with the use of the online service MultAlin (http://multalin.toulouse.inra.fr/multalin/).** The SNPs are colored in blue.

**References**

1. Feodorova VA, Zaitsev SS, Khizhnyakova MA, Saltykov YV, Evstifeev VV, Khusainov FM, Yakovlev SI, Larionova OS, Motin VL. Data of *de novo* genome assembly of the *Chlamydia psittaci* strain isolated from the livestock in Volga Region, Russian Federation. Data Brief. 2020; 29:105190.

2. Feodorova VA, Saltykov YV, Kolosova AA, Evstifeev VV, Khusainov FM, Ivanova SV, Morozova DD, Yakovlev SI, Larionova OS. Russian vaccines against chlamydia infection in livestock. Acta Vet. 2023.

3. Zaręba-Marchewka K, Szymańska-Czerwińska M, Livingstone M, Longbottom D, Niemczuk K. Whole Genome Sequencing and Comparative Genome Analyses of *Chlamydia abortus* Strains of Avian Origin Suggests That *Chlamydia abortus* Species Should Be Expanded to Include Avian and Mammalian Subgroups. Pathogens. 2021;10(11):1405.

4. Pannekoek Y, Dickx V, Beeckman DS, Jolley KA, Keijzers WC, Vretou E, et al. Multi locus sequence typing of *Chlamydia* reveals an association between *Chlamydia psittaci* genotypes and host species. PLoS One. 2010; 5:e14179.

5. Longbottom D, Livingstone M, Ribeca P, Beeckman DSA, van der Ende A, Pannekoek Y, Vanrompay D. Whole genome *de novo* sequencing and comparative genomic analyses suggests that *Chlamydia psittaci* strain 84/2334 should be reclassified as Chlamydia abortus species. BMC Genomics. 2021; 22:159.

6. Kaltenboeck B, Kousoulas KG, Storz J. Structures of and allelic diversity and relationships among the major outer membrane protein (*ompA*) genes of the four chlamydial species. J Bacteriol. 1993; 175:487-502.

7. Vanrompay D, Butaye P, Sayada C, Ducatelle R, Haesebrouck F. Characterization of avian *Chlamydia psittaci* strains using *omp1* restriction mapping and serovar-specific monoclonal antibodies. Comparative Study Res Microbiol. 1997; 148:327-333.

8. Kaltenboeck B, Kousoulas KG, Storz J. Structures of and allelic diversity and relationships among the major outer membrane protein (*ompA*) genes of the four chlamydial species. J Bacteriol. 1993; 175:487-502.

9. Siarkou VI, Vorimore F, Vicari N, Magnino S, Rodolakis A, Pannekoek Y, Sachse K, Longbottom D, Laroucau K. Diversification and Distribution of Ruminant *Chlamydia abortus* Clones Assessed by MLST and MLVA. PLoS One. 2015; 10:e0126433.

10. Seth-Smith HMB, Buso LS, Livingstone M, Sait M, Harris SR, Aitchison KD, et al. European Chlamydia abortus livestock isolate genomes reveal unusual stability and limited diversity, reflected in geographical signatures. BMC Genomics. 2017; 18(1):344.

11. Livingstone M, Wheelhouse N, Ensor H, Rocchi M, Maley S, Aitchison K, et al. Pathogenic outcome following experimental infection of sheep with *Chlamydia abortus* variant strains LLG and POS. PLoS ONE. 2017; 12:e0177653.

12. Ruiz-Laiton A, Molano-Ayala N, García-Castiblanco S, Puentes-Orozco AM, Falla AC, Camargo M, et al. The prevalence of *Chlamydia psittaci* in confiscated Psittacidae in Colombia. Prev Vet Med. 2022; 200:105591.

13. Thomson NR, Yeats C, Bell K, Holden MT, Bentley SD, Livingstone M, et al. The Chlamydophila abortus genome sequence reveals an array of variable proteins that contribute to interspecies variation. Genome Res. 2005; 15(5):629-640.

14. Rodolakis A, Souriau A. Response of ewes to temperature sensitive mutants of *Chlamydia psittaci* (var. ovis) obtained by NTG mutagenesis. Ann Rech Vet. 1983; 14:155–161.

15. Rodolakis A, Bernard F, Souriau A. Stability and safety of a chlamydial live vaccine against enzootic abortion of ewes. Vaccine. 1989; 7(2):178.

16. Rodolakis A, Souriau A. Response of goats to vaccination with temperature-sensitive mutants of *Chlamydia psittaci* obtained by nitrosoguanidine mutagenesis. Am J Vet Res. 1986; 47:2627–2631.

17. Longbottom D, Sait M, Livingstone M, Laroucau K, Sachse K, Harris SR, Thomson NR, Seth-Smith HMB. Genomic Evidence That the Live *Chlamydia Abortus* Vaccine Strain 1B Is Not Attenuated and Has the Potential to Cause Disease. Vaccine. 2018; 36:3593–3598.

18. Vanrompay D, Ducatelle R, Haesebrouck F. Pathogenicity for turkeys of Chlamydia psittaci strains belonging to the avian serovars A, B and D. Avian Pathol. 1994; 23:247–262.

19. Van Lent S, Piet JR, Beeckman D, et al. Full genome sequences of all nine *Chlamydia psittaci* genotype reference strains. J Bacteriol. 2012; 194:6930–6931.

20. Voigt A, Schöfl G, Heidrich A, Sachse K, Saluz HP. Full-length de novo sequence of the *Chlamydophila psittaci* type strain, 6BC. J Bacteriol. 2011; 193(10):2662-2663.

21. Voigt A, Schöfl G, Saluz HP. The *Chlamydia psittaci* Genome: A Comparative Analysis of Intracellular Pathogens. PLoS ONE. 2012; 7(4):e35097.

22. Seth-Smith HM, Harris SR, Rance R, West AP, Severin JA, Ossewaarde JM, et al. Genome sequence of the zoonotic pathogen *Chlamydophila psittaci*. J Bacteriol. 2011; 193(5):1282-1283.

23. Sachse K, Hölzer M, Vorimore F, Barf L-M, Lamkiewicz K, Sachs C, Laroucau K, Marz M. Extensive genomic divergence among 61 strains of *Chlamydia psittaci*. bioRxiv. 2022; 11.

24. White RT, Jelocnik M, Klukowski N, Haque MH, Sarker S. The first genomic insight into *Chlamydia psittaci* sequence type (ST) 24 from a healthy captive psittacine host in Australia demonstrates evolutionary proximity with strains from psittacine, human, and equine hosts. Vet Microbiol. 2023; 280:109704.

25. Jelocnik M, Branley J, Heller J, Raidal S, Alderson S, Galea F, Gabor M, Polkinghorne A. Multilocus sequence typing identifies an avian-like *Chlamydia psittaci* strain involved in equine placentitis and associated with subsequent human psittacosis. Emerg Microbes Infect. 2017; 6(2):e7.

26. Schöfl G, Voigt A, Litsche K, Sachse K, Saluz HP. Complete genome sequences of four mammalian isolates of Chlamydophila psittaci. J Bacteriol. 2011; 193(16):4258.

27. Jenkins C, Jelocnik M, Micallef ML, Galea F, Taylor-Brown A, Bogema DR, et al. An epizootic of *Chlamydia psittaci* equine reproductive loss associated with suspected spillover from native Australian parrots. Emerg Microbes Infect. 2018; 7(1):88.

28. Vorimore F, Aaziz R, de Barbeyrac B, Peuchant O, Szymańska-Czerwińska M, Herrmann B, Schnee C, Laroucau K. A New SNP-Based Genotyping Method for *C. psittaci*: Application to Field Samples for Quick Identification. Microorganisms. 2021; 9:625.

29. Shima K, Weber MM, Schnee C, Sachse K, Käding N, Klinger M, Rupp J. Development of a Plasmid Shuttle Vector System for Genetic Manipulation of *Chlamydia psittaci*. mSphere. 2020; 5(4):e00787-20.

30. Seth-Smith HM, Sait M, Sachse K, Gaede W, Longbottom D, Thomson NR. Genome Sequence of Chlamydia psittaci Strain 01DC12 Originating from Swine. Genome Announc. 2013; 1(1):e00078-12.

31. Bedson SP, Bland JOW. A morphological study of psittacosis virus, with the description of a developmental cycle. Br J Exp Pathol. 1932; 13:461–466.

32. Grinblat–Huse V, Drabek EF, Creasy HH, et al. Genome sequences of the zoonotic pathogens *Chlamydia psittaci* 6BC and Cal10*.* J Bacteriol. 2011; 193:4039–4040.
